# From Fermentation to Functionality: Lipidomic Characterization and Index‐Based Evaluation of Plant‐Based Kefir

**DOI:** 10.1002/fsn3.71662

**Published:** 2026-03-24

**Authors:** Buse Usta‐Gorgun, Lutfiye Yilmaz‐Ersan

**Affiliations:** ^1^ Graduate School of Natural and Applied Sciences, Bursa Uludag University Bursa Turkiye; ^2^ Faculty of Agriculture, Department of Food Engineering Bursa Uludag University Bursa Turkiye

**Keywords:** fatty acid, fermentation, kefir, lipid index, plant milk

## Abstract

This study aimed to characterize the fatty acid composition and to evaluate lipid quality indices of kefir produced with varying ratios of chestnut milk and reconstituted skim milk, using both traditional and commercial starter cultures. Lactobacillaceae counts remained high in all samples (8.35–8.70 log cfu/g on MRS and 8.45–9.35 log cfu/g on M17), while intermediate chestnut milk substitution ratios promoted higher yeast growth than either 100% reconstituted or 100% plant‐based formulations, particularly in samples fermented with grains. Chestnut milk samples fermented with kefir grains or commercial starter cultures exhibited distinct fatty acid profiles and changes in lipid quality during storage. The 20% chestnut milk sample had significantly higher levels of butyric, caproic, caprylic, capric, myristic, and palmitic acids and α‐linolenic acid. In contrast, the 100% chestnut milk sample exhibited the highest concentrations of linoleic and γ‐linolenic acids. Fermentation with kefir grains resulted in elevated butyric, caproic, capric, palmitic, margaric, and arachidic acids, as well as polyunsaturated linoleic acid. Samples fermented with a commercial starter culture contained higher levels of oleic, γ‐linolenic, α‐linolenic, nonadecylic, and gadoleic acids. Throughout storage, short‐ and medium‐chain fatty acids and polyunsaturated fatty acids (ω‐6 and ω‐3) decreased, while palmitic, margaric, oleic, and arachidic acids increased. The 20% chestnut milk sample had the highest contents of saturated fatty acids, total ω‐3 fatty acids, and short‐ and medium‐chain fatty acids. The 100% chestnut milk sample had the highest levels of total unsaturated fatty acids, ω‐6 fatty acids, and long‐chain fatty acids. Lipid quality indices were most favorable in the 100% chestnut milk and commercial starter fermentations, showing lower atherogenic and thrombogenic indices. These results highlight chestnut milk‐based kefir as a low‐fat, functional dairy alternative with an enhanced bioactive fatty acid content and favorable lipid quality indices.

## Graphical abstract

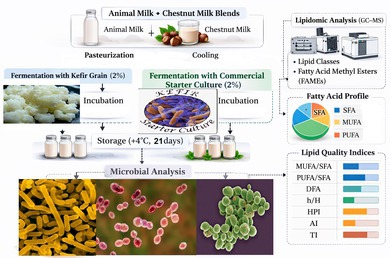

## 1 Introduction

Nowadays, industrialization, urbanization, socioeconomic developments, and market globalization have led to changes in lifestyles and diets. In addition, maintaining an appropriate energy intake and engaging in sufficient physical activity are crucial for overall well‐being and a healthy lifestyle. Dietary fats are key determinants of the nutritional and economic value of both vegetable and animal foods, serving as a principal bioenergetic substrate for tissues. They also actively participate in functions related to the unique sensory and textural properties of foods, which are critical to consumer acceptability.

Fatty acids in dietary fats originate from triglycerides and phospholipids and serve as essential biological molecules involved in nutrition, metabolic processes, physiological function, and overall health maintenance. Fatty acids are organic compounds characterized by the presence of at least one carboxyl group (–C(=O)OH, –COOH, or –CO_2_H) and an extended hydrocarbon chain. This chain may consist of single bonds, as observed in saturated fatty acids (SFAs), or one or more double bonds, as found in unsaturated fatty acids (UFAs). UFAs exhibit distinct geometric isomerism, predominantly in cis and trans configurations. Trans isomers are stereoisomers of cis‐UFAs. The composition and concentration of fatty acids in dietary sources significantly impact their nutritional and therapeutic properties, as well as their sensory appeal to consumers. Accordingly, current research increasingly emphasizes the evaluation of lipid quality indices derived from the fatty acid profiles of various food sources (Abbas et al. 2021; Despal et al. 2021; Turek and Wszołek 2022; Yilmaz Ersan and Suna 2024; Guo et al. 2025; Paszczyk and Tońska 2025). These indices characterize the positive and negative effects of fatty acids consumed via dietary sources on nutritional quality and therapeutic functions. The most commonly used indices are ‘total SFAs’, ‘polyunsaturated fatty acids (PUFAs)’, ‘PUFAs/SFAs ratio’, ‘ω6/ω3’, ‘the atherogenicity index (AI)’, ‘the thrombogenicity index (TI)’, ‘the health‐promoting index (HPI)’, ‘the hypocholesterolemic/hypercholesterolemic ratio (h/H)’, ‘desirable fatty acids (DFA)’, ‘the unsaturation index (UI)’, ‘the total of eicosapentaenoic acid and docosahexaenoic acid (EPA + DHA)’, ‘fish lipid quality/flesh lipid quality ratio (FLQ)’, ‘the linoleic acid/α‐linolenic acid (LA/ALA) ratio’, and total trans fatty acid (TFAs). AI defines some SFAs/UFAs and is linked to the adhesion tendency of fatty acids in the arteries. TI is the relationship between the sum of prothrombogenic SFAs and the sum of anti‐thrombogenic UFAs. Elevated AI values are indicative of atherosclerosis, resulting from the accumulation of fatty acids within cells of the circulatory and immune systems. Similarly, increased TI values indicate a higher propensity for thrombus formation and subsequent development of fatty plaques in blood vessels. Increased AI, TI, and ω6/ω3 ratios have been associated with a higher likelihood of developing chronic disorders such as cardiovascular and autoimmune diseases, as well as cancer and obesity. Generally, the diet recommends a ratio of ω‐6/ω‐3 within the limits of 4–5:1. The h/H is a ratio of hypocholesterolemic FAs (PUFAs and oleic acid)/hypercholesterolemic FAs (C12:0, C14:0, and C16:0), while DFA is the total of UFAs and stearic acid. HPI indicates UFAs/some SFAs (C12:0, C14:0, and C16:0). High h/H, PUFA/SFA, DFA, and HPI values are desirable for the nutritional and therapeutic functions of dietary fatty acids (Chen and Liu 2020; Reis Lima et al. 2020; Yilmaz Ersan and Suna 2024).

The nutritional value of milk is described by protein, fat, lactose, vitamins, and minerals. Milk fat is a value‐added determinant of milk's functional and economic specificity. Milk fat includes lipidomics composed of triacylglycerols or triglycerides, diacylglycerols or diglycerides, glycerophospholipids, sphingolipids, nonesterified fatty acids, cholesterols, and glycolipids. The principal constituents of the milk fat globule membrane (MFGM) are glycerophospholipids, sphingolipids, and glycolipids, which contribute to the stabilization of the oil‐in‐water (o/w) emulsion and exert various health‐promoting effects, including cardioprotective, anticancer, anticholesterolemic, and anti‐inflammatory activities. Milk fat contains more than 400 distinct fatty acids that significantly influence the physicochemical, rheological, organoleptic, and oxidative properties of dairy products, as well as their yield and nutritional quality. Furthermore, it serves as a rich source of lipophilic vitamins, such as β‐carotene, retinol, and α‐tocopherol, which possess potent antioxidant functions (Laučienė et al. 2019; Ozcan and Demiray‐Teymuroglu 2020). Despite the high concentration of SFAs in milk fat causes cardiovascular disease, relevant investigations have concentrated on the functional fatty acids such as butyric acid, oleic acid, vaccenic acid, conjugated linoleic acid, and α‐linolenic acid, which may show health‐promoting effects, including anti‐carcinogenic, anti‐lipogenic, and anti‐inflammatory activity (Yilmaz‐Ersan 2013; Ceylan and Ozcan 2020; Despal et al. 2021; Guo et al. 2025). Kefir is manufactured by fermenting milk with a commercial starter culture or kefir grains, which are composed of various species of lactic acid bacteria, acetic acid bacteria, yeasts, and mycelial fungi. The acid‐alcoholic fermentation of milk with these microorganisms yields a refreshing, slightly acidic, and carbonated beverage. It includes nutrient‐density components (protein, milk fat, minerals, and vitamins), and fermentation leads to the formation of therapeutic compounds such as essential amino acids, peptides, organic acids, and healthy fatty acids. Generally, different animal milks, such as cow, ewe, and goat, are used for kefir production. Recently, plant‐based milks like soy, walnut, hazelnut, peanut, cashew, pumpkin, coconut, and rice have been used for production (Liu et al. 2005; Cui et al. 2013; Botelho et al. 2014; Bensmira and Jiang 2015; Koh et al. 2017; Deeseenthum et al. 2018; Karina et al. 2018; Silva et al. 2018; Atalar 2019; dos Santos et al. 2019; Łopusiewicz et al. 2019; Taleb Abadl et al. 2022; Araujo Filho et al. 2023). The type of milk, starter culture type and quantity, ingredients, fermentation, and storage conditions (including time and temperature) are important discriminators that affect the nutritional, rheological, sensorial, and therapeutic properties of kefir (Ozcan et al. 2019; Yilmaz‐Ersan et al. 2024).

Chestnuts represent a valuable nutritional source, rich in carbohydrates, proteins, and dietary fiber. Despite their low total fat content, they are rich in essential fatty acids, notably linoleic and linolenic acids, which have been linked to the prevention of cardiovascular disease and to supporting neurodevelopment and retinal maturation in infants. Furthermore, chestnuts possess a minor lipid fraction enriched with bioactive, fat‐soluble compounds, such as tocopherols and phytosterols, which contribute to their overall functional and health‐promoting properties (Astorga España et al. 2011; Usta‐Gorgun et al. 2022; Gonçalves et al. 2023). In recent years, with changes in consumer awareness and expectations, demand for healthy, low‐fat food products has increased in the food industry as consumers aim to reduce their fat intake. As consumer demand for low‐ and zero‐fat products increases, efforts to develop them have also increased. Additionally, a well‐balanced, diversified diet is gaining popularity among consumers who are aware of the link between dietary patterns and health outcomes. In this context, incorporating foods from both animal and plant sources into the daily diet significantly increases the intake of healthy bioactive compounds. Chestnuts have the lowest fat among nuts, so in the present study, chestnut milk was chosen as a plant‐based milk. Furthermore, the reconstituted milk, a substitute for animal milk, was prepared from skim milk powder. Kefir with animal and chestnut milk was developed as a novel, low‐fat fermented product. The fatty acid composition of plant milk‐based kefir was analyzed using gas chromatography–mass spectrometry (GC–MS). Lipid quality indices were subsequently computed based on the quantitative distribution of individual fatty acids.

## 2 Materials and Methods

### 2.1 Chestnut Milk

To optimize the production of chestnut‐based plant milk, a Response Surface Methodology (RSM) approach combined with a Central Composite Design was applied across 30 experimental trials. Four key factors were investigated: dilution ratio (33; 25; 20; 17, and 14 g/100 mL), dilution temperature (20°C; 37.5°C; 55°C; 72.5°C and 90°C), pasteurization time (10, 15, 20, 25 and 30 min), and pasteurization temperature (70°C; 75°C; 80°C; 85°C and 90°C). The responses measured included total phenolic content and antioxidant activity, assessed using the 2,2′‐azinobis‐(3‐ethylbenzothiazoline‐6‐sulfonic acid) (ABTS) radical scavenging assay and the ferric reducing antioxidant power (FRAP) assay. By comparing predicted and observed results, the optimal conditions for chestnut milk preparation were established as a 1:4 dilution ratio, with the mixture diluted at 37.57°C and subsequently pasteurized at 84.43°C for 25 min (Usta‐Gorgun et al. 2022).

### 2.2 Reconstituted Skimmed Milk

Skim milk powder was reconstituted in distilled water at a concentration of 10.70% (w/v) to approximate the compositional characteristics of raw skim milk. The reconstituted milk was then heated to 90°C for 10 min.

### 2.3 Kefir Starter Cultures and Activation Procedure

The Food Engineering Department (Bursa Uludag University, Bursa, Turkiye) provided kefir grains containing *Lactobacillus* sp., *Lactococcus* sp., acetic acid bacteria, and yeasts. The commercial kefir starter culture used in this study was Danisco CHOOZIT Kefir DC LYO, manufactured by Danisco Biolacta (Olsztyn, Poland). This lyophilized (LYO) culture was a symbiotic blend of kefir grain microbiota, kefir yeasts, *Lactococcus lactis* subsp., *Leuconostoc* sp., *Lactobacillus* sp., and *Streptococcus thermophilus* . The product was supplied in DVI format with a recommended inoculation rate of one sachet per 1000 L of milk. For kefir grain activation, the grains were placed in sterile jars containing skimmed UHT milk and incubated at 25°C for 18–20 h in an aerobic and static environment. This process was repeated 3 times before the grains were used in production. Commercial kefir starter culture was added to sterile reconstituted milk with a dry matter content of 10.70% ± 0.03% at 25°C, and the culture was incubated at the same temperature in an aerobic, static environment until the pH reached 4.8.

### 2.4 Plant Milk‐Based Kefir Production

**FIGURE 1:**
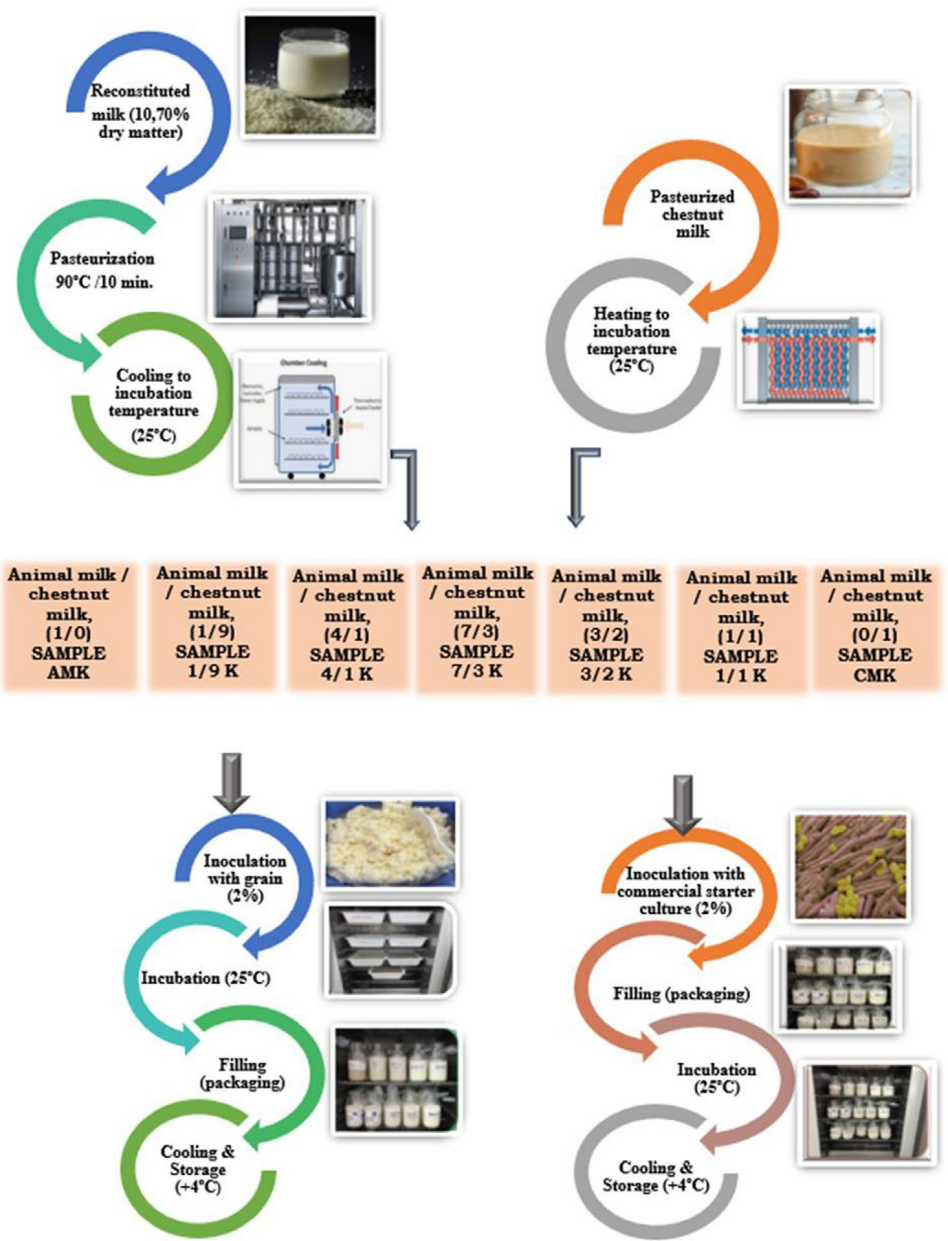
Plant milk‐based kefir production.

Seven ratios of reconstituted skim milk to chestnut milk (Table 1) were prepared by blending the heated skim milk with chestnut milk and adjusting the mixture to 25°C for inoculation. Each milk blend was fermented using traditional kefir grains and a commercial starter culture (2% wt/vol) at 25°C until the pH reached 4.7. Following fermentation, the samples were held at room temperature (20°C ± 1°C) for 30 min before being refrigerated at 4°C ± 1°C for 21 days. The workflow for producing plant milk‐based kefir is illustrated in Figure 1. Fatty acid profiles were analyzed at the start (Day 1) and end (Day 21) of storage.

**TABLE 1:** Product codes.

Product code	Reconstituted skim milk (%)	Chestnut milk (%)
AMK‐100:0	100	0
1/9K‐90:10	90	10
4/1K‐80:20	80	20
7/3K‐70:30	70	30
3/2K‐60:40	60	40
1/1K‐50:50	50	50
CMK‐0:100	0	100

### 2.5 Microbiological Assay

Enumeration of Lactobacillaceae was performed using MRS and M17 agar media (Merck, Germany). For this purpose, MRS and M17 plates were incubated at 30°C for 72 h under anaerobic conditions established using the Anaerocult C system (Merck, Germany) (Irigoyen et al. 2005). Yeast counts were assessed separately on Yeast Glucose Chloramphenicol Agar (YGCA) (Condalab, Spain) following aerobic incubation at 25°C for 3–5 days. The incubation regimes and counting procedures were selected in accordance with previously validated methodologies and international standard guidelines (Witthuhn et al. 2005).

### 2.6 Total Fat Content Assay

The Soxhlet extraction technique was performed for total fat analysis. Two grams of a sample using hexane as a solvent were extracted in Solvent Extractors (SER‐148 Series, VELP‐Scientifica, Italy). Approximately 4–5 g of the dried sample was weighed into small, tared filter bags. The mouths of the filter bags were sealed with a heat sealer, spaced approximately 4 mm apart. Sample bags were dried in an oven at 105^o^C for 1 h, cooled in a dessicator, and subsequently weighed. The weighed bags were placed in the device, and hexane was used as the solvent for oil extraction at 130°C ± 2°C. After the 6‐h extraction, the hexane was removed, and the extracts were dried at 105°C for 60 min. Subsequently, the extracts were allowed to cool to room temperature, and weighing was continued until a constant mass was obtained. The fat content of the samples was then determined gravimetrically and expressed as grams per 100 mL (AOAC 2000; VELP Scientifica 2018).

### 2.7 Fatty Acid Composition Assay

**FIGURE 2:**
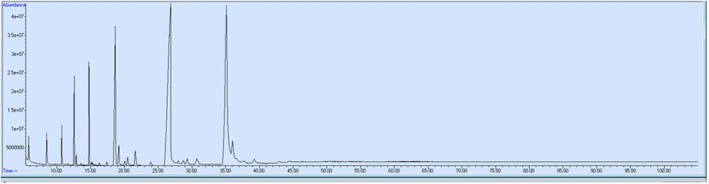
Chromatogram of the fatty acid profile.

Fatty acid standards with carbon numbers ranging from C4 to C20 were obtained from Sigma‐Aldrich (Dublin, Ireland). The extraction of lipids in the kefir samples was performed according to AOAC 963.22, and the fatty acid methyl esters (FAMEs) were prepared based on the IUPAC method II.D.19. Lipids were extracted from the samples using 20 mL of chloroform: methanol (2:1, v/v) per sample under vortex mixing for 30–60 s to ensure complete homogenization. To facilitate phase separation, the mixture was vortexed again for 30 s and allowed to stand until two phases formed. The lower chloroform‐rich phase, containing the lipids, was carefully collected and transferred to a clean tube. The chloroform: methanol solvent mixture was then removed using a rotary vacuum evaporator (Laborota 4001, Germany) at 30°C–40°C under reduced pressure, and the lipid extracts were collected. The concentrations of fatty acids were analyzed following their conversion to FAMEs. FAMEs were prepared through transesterification using 1.5 M HCl in methanol, with 100 μL of oil subjected to shaking for 15 min. The methylation process was performed at 80°C for 2 h, after which 1 mL of hexane was added to extract the FAMEs. These were subsequently analyzed using a Gas Chromatography–Mass Spectrometry (GC–MS) system (Agilent Technologies, USA) equipped with a mass spectrometric detector (Agilent Technologies, Japan) and a DB‐WAX capillary column (Agilent Technologies, USA). The experimental parameters are presented in Table 2. Fatty acid peak areas were identified by matching their retention times with those of corresponding standard compounds, which were analyzed under identical conditions. Retention times of fatty acid standards used for chromatographic identification were as follows: butyric acid (5.9 min), caproic acid (8.4 min), caprylic acid (10.6 min), capric acid (12.4 min), lauric acid (14.4 min), myristic acid (18.4 min), palmitic acid (26.1 min), palmitoleic acid (27.3 min), margaric acid (28.0 min), stearic acid (30.1 min), oleic acid (34.3 min), linoleic acid (35.1 min), arachidic acid (36.3 min), γ‐linolenic acid (38.6 min), α‐linolenic acid (39.0 min), and gadoleic acid (42.2 min). The chromatogram of the fatty acid profile is presented in Figure 2. The fatty acid composition of each sample was expressed as the relative percentage of total fatty acids (Bardakci and Secilmis 2006).

**TABLE 2:** Experimental parameters for fatty acid analysis.

*GC conditions*	
GC system	AGILENT 7890A GC
Column	DB WAX (50 × 0.20 mm, 0.20 μm)
Carrier gas	Helium
Temperature gradient	Initial 60°C, 13°C/min to 175°C, hold 27 min 4°C/min to 215°C, hold 5 min 4°C/min to 240°C, hold 15 min
Injection type	Solit mode (20:1)
Injector temperature	240°C
Injection volume	1 μL
Transfer line temperature	230°C
*MS Conditions*	
MS system	AGILENT 5975 MSD—Quadrupole Mass Spectrometry
Mode of operation	SCAN
Cone voltage	60 eV
Source temperature	230°C
Acquisition range	50–500 m/z
Data management	MSDCHEM

### 2.8 Calculation of the Lipid Quality Indices

The indices used to assess the effect of fatty acids on nutritional quality were calculated.

Polyunsaturated fatty acid/saturated fatty acid (PUFA/SFA) = ∑PUFA/∑SFA (Bodnár et al. 2021).

Desirable fatty acids (DFA) = ΣMUFA + ΣPUFA + C18:0 (Barać et al. 2018).

Atherogenicity Index (AI) = [C12:0 + (4 × C14:0) + C16:0]/∑UFA (Bodnár et al. 2021).

Thrombogenicity Index (TI) = (C14:0 + C16:0 + C18:0)/[(0.5 × ∑MUFA) + (0.5 × ∑n‐6 PUFA) + (3 × ∑n‐3 PUFA) + (n‐3/n‐6)] (Bodnár et al. 2021).

Hypocholesterolemic/hypercholesterolemic (h/H) = (cis‐C18:1 + ∑PUFA)/(C12:0 + C14:0 + C16:0) (Bodnár et al. 2021).

Health‐promoting index (HPI) = ∑UFA/[C12:0 + (4 × C14:0) + C16:0] (Bodnár et al. 2021).

### 2.9 Statistical Analysis

Statistical analyses were performed using a three‐factor randomized complete block design. The factors were evaluated separately: (i) the product variety; chestnut milk blending ratios, irrespective of starter culture or storage time, (ii) the culture type; grain or commercial, irrespective of blending ratio or storage time, and (iii) the storage time; irrespective of blending ratio or starter culture. This approach enabled independent analysis of each factor, allowing a clear determination of differences among groups. Variance analysis (ANOVA) at the 1% and 5% significance levels, using Fisher's multiple‐range test (ANOVA), was performed. Distinct letters indicate values that differ significantly from each other at a statistical level. Principal Component Analysis (PCA) was performed in Statistica (StatSoft, USA) to evaluate the relationships among samples based on their microbial counts, fatty acid profiles, lipid quality indices, and storage days.

## 3 Results and Discussion

### 3.1 Microbiological Properties of the Samples

The effects of product formulation, culture type, and storage time on Lactobacillaceae and yeast counts are presented in Table 3. Statistical analysis indicated that Lactobacillaceae counts on MRS and M17 agar were not significantly affected by product variety, maintaining stable populations across all chestnut milk substitution ratios. Lactobacillaceae counts on MRS agar ranged from 8.35 to 8.70 log cfu/g, while Lactobacillaceae counts on M17 agar varied between 8.45 and 9.35 log cfu/g. This stability is consistent with studies on other plant‐based dairy analogues. In line with previous studies, the incorporation of plant‐based or mixed dairy–plant matrices into kefir systems does not necessarily compromise Lactobacillaceae viability when appropriate fermentation conditions are maintained (Fiorda et al. 2017; Bourrie et al. 2016). Similarly, the stability of Lactobacillaceae has been reported in kefir and fermented milk products enriched with oat, soy, almond, and chestnut matrices, where buffering capacity and available carbohydrates supported lactic fermentation without inhibitory effects (Mårtensson et al. 2001; Bernat et al. 2015; Yilmaz‐Ersan et al. 2023). In contrast to the Lactobacillaceae, yeast counts were significantly influenced by product variety, culture type, and storage time. The highest yeast count was recorded in the 1:1 K formulation (50% reconstituted milk plus 50% chestnut milk; 5.56 log cfu/g). Interestingly, intermediate substitution ratios promoted higher yeast growth than 100% reconstituted milk (AMK; 4.64 log cfu/g) or 100% chestnut milk (CMK; 4.45 log cfu/g). Similar trends were observed in mixed plant–dairy fermentations, where intermediate substitution ratios promoted yeast growth more effectively than either full dairy or full plant‐based matrices (Bernat et al. 2015; Fiorda et al. 2017). This behavior has been attributed to the presence of fermentable sugars and accessible nitrogen sources in plant‐based milks, which stimulate yeast metabolism and favor yeast–Lactobacillaceae coexistence in mixed fermentations (Coda et al. 2012). Samples fermented with traditional kefir grains exhibited significantly higher yeast counts (5.55 log cfu/g) than those with commercial starters (3.98 log cfu/g). This finding is consistent with the complex, yeast‐rich microbiota of traditional kefir grains, which are composed of diverse consortia of lactic acid bacteria and yeasts embedded in a polysaccharide matrix (Garrote et al. 2001; Prado et al. 2015). In contrast, commercial starter cultures are generally designed to ensure process standardization and Lactobacillaceae dominance, thereby reducing yeast proliferation, as previously reported for controlled kefir fermentations (Granato et al. 2010). Storage time further modulated yeast dynamics, with a significant increase observed from Day 1 (3.38 log cfu/g) to Day 21 (6.14 log cfu/g). In agreement with earlier studies on kefir and plant‐based fermented beverages, kefir‐associated yeasts exhibit strong adaptability to acidic and refrigerated conditions, allowing continued growth during storage, whereas Lactobacillaceae populations remain relatively stable due to acid stress and nutrient limitation (Magalhães et al. 2011; Bourrie et al. 2016; Mårtensson et al. 2001). Such post‐acidification yeast activity has been linked to aroma development, mild carbonation, and enhanced sensory complexity in kefir and kefir‐like beverages (Guzel‐Seydim et al. 2000). The culture × storage time interaction significantly affected all microbial groups, whereas the three‐way interaction was significant only for yeast. This suggests that while the chestnut milk modulates the initial yeast load, the long‐term microbial stability is primarily governed by the starter culture's ecology. Yeasts in kefir‐like beverages contribute to the production of ethanol and precursors for flavor‐active fatty acids (Guzel‐Seydim et al. 2000). Comparable interaction patterns have been reported in mixed fermented beverage systems, where starter culture characteristics outweigh matrix composition in determining microbial stability (Ranadheera et al. 2012; Bernat et al. 2015).

**TABLE 3:** The microbiological counts of the samples.

Product varieties	Lactobacillaceae on MRS agar	Lactobacillaceae on M17 agar	Yeast
AMK	8.55^a^	9.35^a^	4.64^c^
1:9K	8.47^a^	8.80^a^	4.56^cd^
4:1K	8.57^a^	9.22^a^	4.49^cd^
7:3K	8.70^a^	9.16^a^	4.27^e^
3:2K	8.39^a^	8.74^a^	5.36^b^
1:1K	8.35^a^	8.45^a^	5.56^a^
CMK	8.53^a^	8.86^a^	4.45^d^
*Culture types*			
Grain	8.65^a^	8.98^a^	5.55^a^
Commercial	8.37^a^	8.90^a^	3.98^b^
*Storage days*			
1st day	8.69^a^	8.94^a^	3.38^b^
21st day	8.33^a^	8.94^a^	6.14^a^
*ANOVA*			
Product variety	ns	ns	**
Culture type	ns	ns	**
Storage days	ns	ns	**
Product variety × Culture type	ns	ns	**
Culture type × Storage day	**	**	**
Product variety × Storage day	ns	ns	**
Product variety × Culture type × Storage day	ns	ns	**

*Note:* Lowercase letters indicate significant differences between groups. AMK—100% reconstituted milk; 1:9K—90% reconstituted milk plus 10% chestnut milk; 4:1K—80% reconstituted milk plus 20% chestnut milk; 7:3K—70% reconstituted milk plus 30% chestnut milk; 3:2K—60% reconstituted milk plus 40% chestnut milk; 1:1K—50% reconstituted milk plus 50% chestnut milk; CMK—100% chestnut milk; ns, non‐significant *p* > 0.05.

** *p* ≤ 0.01.

### 3.2 Total Fat Content of the Samples

The total fat contents of the samples are presented in Table 4. Some differences (*p* ≤ 0.01) in the fat contents of samples were statistically determined depending on the ratio of chestnut milk. Differences in culture types and storage days were statistically insignificant (*p* > 0.05). The lowest fat content (0.16%) was observed in the AMK sample (with 100% reconstituted milk), whereas the CMK sample (with 100% chestnut milk) had the highest fat content (0.41%). This increase observed with the addition of chestnut milk can be explained by chestnuts (especially in the dried form) having a higher lipid profile than reconstituted skimmed milk powder. It is known in the literature that chestnut lipids consist largely of UFAs (such as linoleic and oleic acid). This suggests that the addition not only increases the product's total fat content but also alters its fatty acid composition, favoring plant‐derived lipids. The values of the present study (0.16%—0.41%) are lower than the range of 0.93%—2.20% reported by Kasapidou et al. (2023) for almond, oat, and peanut milks, and lower than those reported by Pointke et al. (2022) and Antunes et al. (2024) for soy and hazelnut milks. The main reason for this difference is that the base materials used in our study (skimmed milk powder and low‐lipid chestnuts) have lower natural fat content than those of other oilseeds and nuts. Especially when compared to traditional products, where cow's milk (3.5% fat) is dominant, chestnut milk enrichment can offer a significant alternative in the ‘low‐fat/light’ product segment. Generally, chestnut milk enrichment resulted in higher total fat values across samples. The interaction of ‘culture type × storage day’ was statistically significant. Other interactions were insignificant. However, the significant ‘culture type × storage day’ interaction indicates that the metabolic activity (e.g., lipolytic activity) exhibited by microorganisms throughout storage differs depending on the starting culture. This is a critical finding for maintaining the textural and sensory stability of the fermented product throughout its shelf life. Such interactions may involve pH‐driven structural rearrangements, microbial exopolysaccharide production, or lipid–protein–polysaccharide associations, which can influence product stability and mouthfeel without substantially altering total fat content. Taken together, these findings demonstrate that chestnut milk enrichment supports the development of a nutritionally low‐fat fermented product, while textural stability and sensory performance are mainly governed by microbially driven interactions within the food matrix rather than by changes in total lipid content.

**TABLE 4:** The total fat content of the samples.

*Product varieties*	g 100 mL^−1^
AMK	0.16^d^
1:9K	0.22^cd^
4:1K	0.24^cd^
7:3K	0.25^bcd^
3:2K	0.28^bc^
1:1K	0.33^ab^
CMK	0.41^a^
*Culture types*	
Grain	0.28^a^
Commercial	0.25^a^
*Storage days*	
1st day	0.26^a^
21st day	0.27^a^
*ANOVA*	
Product variety	**
Culture type	ns
Storage days	ns
Product variety × Culture type	ns
Culture type × Storage day	**
Product variety × Storage day	ns
Product variety × Culture type × Storage day	ns

*Note:* Lowercase letters indicate significant differences between groups. AMK—100% reconstituted milk, 1:9K—90% reconstituted milk plus 10% chestnut milk; 4:1K—80% reconstituted milk plus 20% chestnut milk; 7:3K—70% reconstituted milk plus 30% chestnut milk; 3:2K—60% reconstituted milk plus 40% chestnut milk; 1:1K—50% reconstituted milk plus 50% chestnut milk; CMK—100% chestnut milk; ns, non‐significant *p* > 0.05.

** *p* ≤ 0.01.

### 3.3 Fatty Acid Profile of the Samples

The fatty acid profile (%) of the samples is displayed in Table 5. The results are presented as the mean product variety (chestnut milk ratio), starter culture type (grain and commercial), and storage days. 17 fatty acids were analyzed in the samples on the 1st and 21st day of storage. In general, the chestnut milk ratio among the investigated parameters significantly affected the fatty acid profiles of the samples. Differences among samples were statistically significant (*p* ≤ 0.01) in the fatty acid profiles except for stearic acid (C18:0; *p* > 0.05). The sample with 20% chestnut milk (4:1K) had significantly higher mean values for butyric (C4:0), caproic (C6:0), caprylic (C8:0), capric (C10:0), lauric (C12:0), myristic (C14:0), palmitic (C16:0), and α‐linolenic acid (C18:3n‐3; ω‐3) acids. Palmitoleic (C16:1), arachidic (C20:0), and gadoleic (C20:1) acids were the major fatty acids in the sample (7:3K), including chestnut milk 30%. Oleic acid (C18:1) was the highest value in the sample (1:9K) with chestnut milk 10%, whereas nonadecylic acid (C19:0) was in the sample (AMK) with animal milk 100%. Palmitic acid (C16:0), one of the major SFAs in milk fat, has been reported to exhibit strong antimutagenic properties, providing protection against various mutagens (Vieira et al. 2015). Therefore, palmitic acid may play an important role in increasing the antimutagenic potential of fermented milks. The sample with chestnut milk 100% (CMK) had the highest linoleic (C18:2n‐6; ω‐6) and γ‐linolenic (C18:3n6; ω‐6) acid values, which are ω‐6 fatty acids classified as essential fatty acids. In agreement with our findings, Kasapidou et al. (2023) detected that palmitic acid (C16:0) is the major SFA in almond, oat, coconut, and peanut‐based milks. Abbas et al. (2021) reported that palmitic (C16:0), oleic (C18:1), α‐linolenic acid (C18:3n‐3; ω‐3), and linoleic (C18:2n‐6; ω‐6) acids were detected as major fatty acids in the processed cheeses with enriched walnuts. They stated that the walnut addition increased the levels of some fatty acids (C16:1, C18:1, C18:2, and C18:3) in cheese samples. Ventura et al. (2025) reported that the dominant fatty acids in commercial kefir, including plain and flavored varieties (strawberry, blueberry, and apricot), were identified as C18:3, C18:2, C18:1 (comprising both oleic and vaccenic acids), C18:0 (stearic acid), C16:0 (palmitic acid), and C14:0 (myristic acid). Regarding the culture type used for kefir production, there were significant differences in the level of fatty acid concentration except for caprylic (C8:0), lauric (C12:0), myristic (C14:0), and stearic (C18:0) acids. The samples fermented with kefir grain had the higher butyric (C4:0), caproic (C6:0), capric (C10:0), palmitic (C16:0), palmitoleic (C16:1), margaric (C17:0), linoleic (C18:2n‐6; ω‐6) and arachidic (C20:0) acids, while oleic acid (C18:1), γ‐linolenic (C18:3n6; ω‐6), α‐linolenic acid (C18:3n‐3; ω‐3), nonadecylic acid (C19:0) and gadoleic (C20:1) acids were the major fatty acids in samples fermented with a commercial starter culture. Differences among storage days were statistically detected in the fatty acid profiles except for lauric (C12:0) and palmitoleic (C16:1) acids. During storage time, the values of butyric (C4:0), caproic (C6:0), caprylic (C8:0), capric (C10:0), myristic (C14:0), stearic (C18:0), linoleic (C18:2n‐6; ω‐6), γ‐linolenic (C18:3n6; ω‐6), α‐linolenic acid (C18:3n‐3; ω‐3), nonadecylic acid (C19:0) and gadoleic (C20:1) acids decreased, whereas those of palmitic (C16:0), margaric (C17:0), oleic (C18:1), and arachidic (C20:0) acids increased. The interactions of ‘product variety × culture type’, ‘culture type × storage day’, ‘product variety × storage day’ and product variety × culture type × storage day were determined statistically significant for caproic (C6:0), caprylic (C8:0), lauric (C12:0), palmitic (C16:0), linoleic (C18:2n‐6; ω‐6), γ‐linolenic (C18:3n6; ω‐6), α‐linolenic acid (C18:3n‐3; ω‐3), arachidic (C20:0) and gadoleic (C20:1) acids. In a recent study, the polar lipid composition of almond, coconut, and soy beverages was comprehensively characterized and compared with bovine milk using liquid chromatography–quadrupole time‐of‐flight mass spectrometry. A total of 30 polar lipid subclasses and 572 molecular species were identified, primarily belonging to glycerophospholipids and sphingolipids. Coconut milk was distinguished by its abundance of sphingolipids (hexosylceramides and sulfatides), soy milk was particularly rich in phospholipid species, and almond milk predominantly contained fatty acids with 16–18 carbon chains. In contrast, bovine milk contained numerous sphingomyelin species absent in plant‐based beverages (Blasi et al. 2025). The enrichment of short and medium‐chain fatty acids (SCFA and MCFA) observed in formulations with low chestnut milk ratios confirms that milk fat is the primary lipid source in the system and reflects the selective activity of microbial lipase on milk‐derived fatty acids, especially in samples fermented with kefir grains. In contrast, the increased concentrations of oleic, linoleic, and linolenic acids with chestnut milk substitution demonstrate the successful integration of plant lipid fractions into the matrix and the unique stability of these UFAs within the fermented system. The statistically significant ‘product variety × culture type × storage day’ interaction reveals that fatty acid stability is not merely a time‐dependent change but is actively modulated by the specific metabolic pathways of the kefir microbiota (grains and commercial cultures). The higher preservation rates of essential α‐linolenic, γ‐linolenic, and oleic acids in samples fermented with commercial culture can be attributed to lower oxidative degradation rates or to different substrate specificity exhibited by enzyme systems against chestnut‐derived triglycerides. The divergent fatty acid profiles observed between grain and commercial starter cultures demonstrate that microbial consortium complexity plays a determinative role in lipid remodeling, a process shaped by strain‐specific enzymatic activities and microinteractions within the protein‐polysaccharide network. Changes during storage suggest that lipid distribution is governed by gradual matrix reorganization rather than by uncontrolled degradation. Consequently, while the addition of chestnut milk brings the product closer to a lipid profile compliant with cardiovascular health criteria, it simultaneously builds a functional, complex biochemical matrix that supports microbial stability throughout shelf life.

**TABLE 5:** The fatty acid profile (%) of the samples.

Chemical formula	C4: 0 CH_3_ (CH_2_)_2_COOH	C6:0‐CH_3_ (CH_2_)_4_ COOH	C8:0‐CH_3_ (CH_2_)_6_ COOH	C10:0‐CH_3_ (CH_2_)_8_COOH	C12:0‐CH_3_ (CH_2_)_10_COOH	C14:0‐CH_3_ (CH_2_)_12_COOH
Systematic name	Butanoic acid	Hexanoic acid	Octanoic acid	Decanoic acid	Dodecanoic acid	Tetradecanoic acid
Common name	Butyric acid	Caproic acid	Caprylic acid	Capric acid	Lauric acid	Myristic acid
*Product varieties*						
AMK	0.17^c^	0.42^b^	0.81^c^	0.92^bc^	1.34^b^	6.18^b^
1:9K	0.18^c^	0.36^c^	0.65^de^	0.68^d^	1.08^cd^	3.96^de^
4:1K	0.34^a^	0.62^a^	0.99^a^	1.23^a^	1.63^a^	6.74^a^
7:3K	0.18^c^	0.44^b^	0.90^b^	0.99^b^	1.24^bc^	4.75^c^
3:2K	0.28^b^	0.34^c^	0.67^d^	0.75^cd^	0.91^de^	4.11^d^
1:1K	0.18^c^	0.37^c^	0.58^f^	0.59^d^	0.83^e^	3.78^e^
CMK	0.16^c^	0.30^d^	0.64^e^	0.74^cd^	1.00^de^	4.17^d^
*Culture types*						
Grain	0.27^a^	0.45^a^	0.75^a^	0.92^a^	1.14^a^	4.78^a^
Commercial	0.17^b^	0.36^b^	0.75^a^	0.76^b^	1.15^a^	4.84^a^
*Storage days*						
1st day	0.23^a^	0.44^a^	0.81^a^	0.91^a^	1.14^a^	5.07^a^
21st day	0.19^b^	0.38^b^	0.69^b^	0.78^b^	1.15^a^	4.55^b^
*ANOVA*						
Product variety	**	**	**	**	**	**
Culture type	**	**	ns	**	ns	ns
Storage day	**	**	**	*	ns	**
Product variety × Culture type	**	**	**	*	**	**
Culture type × Storage day	*	**	**	ns	*	ns
Product variety × Storage day	**	**	**	*	**	**
Product variety × Culture type × Storage day	ns	**	**	ns	**	**

Chemical formula	C16:0‐CH_3_ (CH_2_)_14_ COOH	C16:1Δ9c (C_16_H_30_O_2_)	C17:0‐(C_17_H_34_O_2_) CH_3_ (CH_2_)_15_CO_2_H	C18:0‐(C_18_H_36_O_2_) CH_3_ (CH_2_)_16_COOH	C18:1; Δ^9^; ω‐9‐(C_18_H_34_O_2_)	C18:2n‐6; *ω‐6*‐(C_18_H_30_O_2_)
Systematic name	Hexadecanoic acid	*cis*‐9‐hexadecenoic acid	Heptadecanoic acid	Octadecanoic acid	*cis*‐9‐octadecenoic acid	*cis*‐9, *cis*‐12‐octadecadienoic acid
Common name	Palmitic acid	Palmitoleic acid	Margaric acid	Stearic acid	Oleic acid	Linoleic acid
*Product varieties*						
AMK	21.64^d^	0.17^b^	0.21^cd^	1.36^a^	28.97^b^	32.19^e^
1:9K	22.56^c^	0,13^c^	0.46^a^	1.30^a^	29.41^a^	31.99^e^
4:1K	27.87^a^	0.15^b^	0.31^b^	1.24^a^	28.93^b^	25.45^f^
7:3K	21.33^d^	0.22^a^	0.25^bc^	1.24^a^	27.13^de^	35.77^c^
3:2K	24.15^b^	0.13^c^	0.14^d^	1.08^a^	27.05^e^	35.44^d^
1:1K	22.88^c^	0.16^b^	0.45^a^	1.07^a^	28.08^c^	36.66^b^
CMK	19.4^e^	0.13^c^	0.24^bc^	1.37^a^	27.40^d^	39.43^a^
*Culture types*						
Grain	23.22^a^	0.17^a^	0.37^a^	1.23^a^	27.69^b^	34.22^a^
Commercial	22.45^b^	0.15^b^	0.21^b^	1.24^a^	28.58^a^	33.29^b^
*Storage days*						
1st day	21.59^b^	0.15^a^	0.26^b^	1.35^a^	27.78^b^	34.40^a^
21st day	24.08^a^	0.15^a^	0.32^a^	1.12^b^	28.50^a^	33.29^b^
*ANOVA*						
Product variety	**	**	**	ns	**	**
Culture type	**	**	**	ns	**	**
Storage day	**	ns	**	*	**	**
Product variety × Culture type	**	**	**	ns	**	**
Culture type × Storage day	**	ns	ns	ns	ns	**
Product variety × Storage day	**	**	**	ns	**	**
Product variety × Culture type × Storage day	**	**	**	ns	**	**

Chemical formula	C18:3‐∆6,9,12‐(18:3n‐6) (C_18_H_30_O_2_) (GLA; ω‐6)	C18:3‐∆9,12,15‐(18:3n‐3) (C_18_H_30_O_2_) (ALA; ω‐3)	C19:0‐(C_19_H_38_O_2_)	C20:0‐(C_20_H_40_O_2_)	C20:1‐Δ9c (C_20_H_38_O_2_)
Systematic name	*cis*‐6, *cis*‐9, *cis*‐12‐octadecatrienoic acid	*cis*‐9, *cis*‐12, *cis*‐15‐octadecatrienoic acid	Nonadecanoic acid	Eicosenoic acid	*cis*‐9‐eicosenoic acid
Common name	γ –Linolenic acid	α‐Linolenic acid	Nonadecylic acid	Arachidic acid	Gadoleic acid
*Product varieties*					
AMK	1.90^bc^	0.31^cd^	0.92^a^	0.21^b^	0.38^b^
1:9K	1.96^b^	0.33^c^	0.64^bc^	0.24^b^	0.33^c^
4:1K	1.10^e^	0.41^a^	0.76^ab^	0.29^a^	0.38^b^
7:3K	1.63^d^	0.37^b^	0.87^a^	0.32^a^	0.51^a^
3:2K	1.71^cd^	0.36^b^	0.52^cd^	0.24^b^	0.49^a^
1:1K	2.00^b^	0.29^d^	0.43^d^	0.32^a^	0.32^c^
CMK	2.31^a^	0.32^c^	0.78^ab^	0.22^b^	0.37^b^
*Culture types*					
Grain	1.68^b^	0.32^b^	0.65^b^	0.28^a^	0.35^b^
Commercial	1.92^a^	0.36^a^	0.75^a^	0.24^b^	0.44^a^
*Storage days*					
1st day	1.95^a^	0.40^a^	0.83^a^	0.23^b^	0.45^a^
21st day	1.64^b^	0.28^b^	0.58^b^	0.28^a^	0.34^b^
*ANOVA*					
Product variety	**	**	**	**	**
Culture type	**	**	*	**	**
Storage day	**	**	**	**	**
Product variety × Culture type	**	**	**	**	**
Culture type × Storage day	**	**	ns	*	**
Product variety × Storage day	**	**	**	**	**
Product variety × Culture type × Storage day	**	**	ns	**	**

*Note:* Lowercase letters indicate significant differences between groups.

Abbreviations: AMK—100% reconstituted milk, 1:9 K–90% reconstituted milk plus 10% chestnut milk; 4:1 K–80% reconstituted milk plus 20% chestnut milk; 7:3 K—70% reconstituted milk plus 30% chestnut milk; 3:2 K—60% reconstituted milk plus 40% chestnut milk; 1:1 K—50% reconstituted milk plus 50% chestnut milk; CMK—100% chestnut milk; ns, non‐significant *p* > 0.05.

** *p* ≤ 0.01.

* *p* ≤ 0.05.

Table 6 summarizes the fatty acid profile, showing that 11 fatty acids identified in the present study were saturated, whereas six were unsaturated. As with the description of fatty acid composition, significant differences were detected (*p* ≤ 0.01) among product varieties depending on the chestnut milk ratio. The highest levels of SFA, total ω3, SCFA, and MCFA were determined in the sample (4:1K) with 20% chestnut milk. The sample (CMK) with 100% chestnut milk had the highest total PUFA, total UFA, total ω‐6, and LCFA. These findings are consistent with the fatty acid profile of chestnuts, which is rich in UFAs, with linoleic, oleic, and palmitic acids. Similarly, saturated fat values were determined as 0.21 g for almond milk, 2.09 g for coconut milk, 0.14 g for soy milk, 0.16 g for oat milk, 0.17 g for rice milk, and 0.51 g for cashew milk (Gómez Franco 2025). In their study on plant‐based milk alternatives, Pointke et al. (2022) found that SFA values varied among different types, ranging from 0.1 to 0.3 g/100 g in almond‐based milk, 0.1 to 0.5 g/100 g in oat‐based milk, and 0.2 to 0.4 g/100 g in soy‐based milk. Martínez‐Padilla et al. (2020) reported that coconut‐based beverages exhibited the highest proportion of SFA, mainly lauric (C12:0) and myristic (C14:0) acids, while other plant‐based milk alternatives (almond, hazelnut, hemp, oat, quinoa, rice, and soy) contained considerably lower SFA levels. In their study, hazelnut‐based drinks showed the highest MUFA content (80%), and hemp‐based beverages were identified as the richest source of PUFA (74%), with oleic (C18:1) and linoleic (C18:2, ω‐6) acids being the predominant fatty acids. Walther et al. (2022) mentioned that plant‐based drinks, including almond, cashew, coconut, hemp, oat, rice, soy, and spelt drinks, predominantly contain LCFAs, except for coconut‐based drinks, which are richer in short‐ and medium‐chain fatty acids. Coconut drinks exhibited the highest SFA content, whereas other plant‐based milks were mainly composed of MUFA and PUFA. Meiland et al. (2025) stated that, unlike bovine milk, the fat globule membranes in plant‐based beverages are composed of a single phospholipid layer, and the triglyceride core exhibits a markedly different composition, with a higher proportion of long‐chain UFAs. In a recent study, Antunes et al. (2024) reported marked differences in the fatty acid composition of various plant‐based milk alternatives (soya, oat, rice, almond, coconut, and hazelnut) compared with cow's milk. Coconut milk exhibited the highest SFA content (79.1%), exceeding even dairy milk, while other milk alternatives contained markedly lower SFA levels (~14.7%). Hazelnut and almond milks were particularly rich in MUFA, accounting for 79.8% and 67% of total fatty acids, respectively. Conversely, soy and rice milks displayed the highest PUFA levels (~62.3%), while coconut milk had the lowest PUFA content (5.7%). Concerning the culture type, there were significant differences (*p* ≤ 0.01) within the description of fatty acid composition, except for MCFA and LCFA. The samples fermented with kefir grain had the highest total SFA, total PUFA, total ω‐6, and SCFA, whereas the values of total MUFA, total UFA, and total ω‐3 were higher than those of the other. This could be related to the lipolytic activities and catabolic reactions of microorganisms in grain and commercial cultures. It was found that the description of fatty acid composition was influenced by 21‐day storage, except for LCFAs (*p* ≤ 0.01). During storage days, the values of total PUFA, total UFA, total ω‐6, total ω‐3, SCFA, and MCFA decreased, while the values of total SFA and total MUFA increased. In a relevant study, yogurt and probiotic‐fermented milk stored for 21 days showed a slight increase in SFA and a decrease in MUFA and PUFA, whereas kombucha‐fermented milk showed an increase in MUFA and PUFA, accompanied by a slight reduction in SFA (Vukić et al. 2019). The interactions of ‘product variety × culture type’ and ‘product variety × storage day’ were determined to be statistically significant for all the descriptions (*p* ≤ 0.01). Although SFAs, which raise total and low‐density lipoprotein (LDL) cholesterol levels in the blood, are a risk factor for cardiovascular disease, scientific studies have highlighted their individual health benefits. Butyric acid (C4:0) serves as an energy source for colon epithelial cells and exhibits anticarcinogenic properties. Caprylic (C8:0), capric (C10:0), and lauric acid (C12:0) have antimicrobial properties. Caproic (C6:0), caprylic (C8:0), and capric (C10:0) acids can contribute to reducing body fat (González‐Martín et al. 2020). Stearic acid (C18:0) is converted to oleic acid (C18:1; ω9), known as a healthier fatty acid. MUFAs and PUFAs reduce the LDL cholesterol level in blood serum instead of the SFAs. Palmitoleic acid (C16:1) among MUFAs can be formed by desaturation of palmitic acid (C16:0) induced by Δ9‐stearoyl‐ACP desaturase. Palmitoleic acid has been shown to have positive health effects, including the prevention of beta‐cell apoptosis, improved cholesterol metabolism, enhanced hemostasis, and increased insulin sensitivity (Adamska et al. 2017; Barać et al. 2022). Compared to PUFAs, as MUFAs are less sensitive to peroxidation, higher MUFA concentration leads to lower rancidity and a longer shelf‐life in foods. Furthermore, since MCFAs are more hydrophilic than LCFAs, they do not require solubilization as micelles, which necessitates energy as a prerequisite for absorption, and thus exhibit beneficial health effects, such as body weight reduction, treatment of malnutrition, and anti‐inflammatory properties (Yilmaz‐Ersan et al. 2023). Kasapidou et al. (2023) determined that SFA, MUFA, and PUFA values of plant‐based milk alternatives, including almond, oat, coconut, and peanut, ranged from 10.46 to 80.66, from 17.53 to 74.51, and from 1.80 to 46.37, respectively. They highlighted that coconut‐based milk had the highest SFA content and the lowest MUFA and PUFA content. Vieira et al. (2015) examined the proportions of SFA, MUFA, and PUFA in semi‐skimmed milk fermented with different kefir grains ranged between 72.70–88.60 g/100 g, 7.23–25.80 g/100 g, and 1.50–7.99 g/100 g, respectively, and resulted in a significant increase in SFA and a reduction in MUFA compared to unfermented milk. In light of all this data, the transformative effect of increasing the chestnut milk ratio on the lipid profile should be evaluated not merely as a change in the raw material but as a restructuring of the product's biochemical matrix. The concentration of SFA and SCFA levels in samples with low chestnut ratios (20%) confirms the dominant character of animal milk lipids in the system and the preferential lipolytic activity of kefir microflora on these lipids. In contrast, the high PUFA and UFA levels observed in pure chestnut milk samples (CMK) indicate that the UFA character of chestnut was successfully transferred to the fermented system, and these components were preserved despite fermentation stress. The increase in SFA values, coupled with decreases in PUFA and ω‐3/ω‐6 levels during storage, suggests that unsaturated bonds are more susceptible to oxidation and that lipid oxidation products are converted over time to more stable saturated forms or secondary metabolites. In particular, the significance of the ‘product variety × culture type’ interaction suggests that kefir grains and commercial cultures have different enzyme specificities: the grain microbiota appears more effective at breaking down milk fat, while commercial cultures play a more efficient role in stabilizing or transforming plant lipids. From a nutritional physiology perspective, these changes prove that the product is not just a beverage but a functional lipid carrier. The single‐layer phospholipid structure in plant‐based milks, as indicated by Meiland et al. (2025), may increase the absorption rate and bioavailability of PUFAs incorporated into the fermented system with the addition of chestnut milk. Consequently, while chestnut milk enrichment brings the product closer to an LDL cholesterol‐lowering MUFA/PUFA balance, it also enables the presentation of fatty acids such as SCFA and MCFA, which play a critical role in colon health and metabolic rate, within a ‘synergistic matrix’ through kefir fermentation.

**TABLE 6:** Description of fatty acid profile.

	Total SFA	Total MUFA	Total PUFA	Total UFA	Total ω6	Total ω3	SCFA (C0:0 to C5:0)	MCFA (C6:0 to C12:0)	LCFA (C13:0 to C21:0)
*Product varieties*									
AMK	34.20^b^	29.52^b^	34.39^d^	63.91^d^	34.08^d^	0.31^cd^	0.17^c^	4.41^b^	93.50^bc^
1:9K	32.13^cd^	29.87^a^	34.27^d^	64.14^d^	33.95^d^	0.32^c^	0.18^c^	3.43^c^	92.60^c^
4:1K	42.06^a^	29.47^b^	26.96^e^	56.43^e^	26.55^e^	0.41^a^	0.34^a^	5.25^a^	92.90^c^
7:3K	32.53^cd^	27.86^d^	37.77^c^	65.63^c^	37.40^c^	0.37^b^	0.18^c^	4.45^b^	93.50^bc^
3:2K	33.21^bc^	27.66^d^	37.51^c^	65.18^c^	37.15^c^	0.36^b^	0.28^b^	3.20^cd^	94.90^ab^
1:1K	31.50^d^	28.56^c^	38.95^b^	67.50^b^	38.65^b^	0.29^d^	0.18^c^	2.81^d^	96.00^a^
CMK	29.02^e^	27.90^d^	42.06^a^	69.96^a^	41.74^a^	0.32^c^	0.16^c^	3.46^c^	95.30^a^
*Culture types*									
Grain	34.08^a^	28.21^b^	36.22^a^	64.43^b^	35.90^a^	0.32^b^	0.25^a^	3.93^a^	94.30^a^
Commercial	32.96^b^	29.17^a^	35.76^b^	64.93^a^	35.39^b^	0.36^a^	0.17^b^	3.78^a^	93.90^a^
*Storage days*									
1st day	32.89^b^	28.37^b^	36.76^a^	65.14^a^	36.36^a^	0.40^a^	0.23^a^	4.13^a^	93.70^a^
21st day	34.15^a^	29.01^a^	35.22^b^	64.23^b^	34.93^b^	0.28^b^	0.19^b^	3.58^b^	94.50^a^
*ANOVA*									
Product variety	**	**	**	**	**	**	**	**	**
Culture type	**	**	**	**	**	**	**	ns	ns
Storage days	**	**	**	**	**	**	**	**	ns
Product variety × Culture type	**	**	**	**	**	**	**	**	**
Culture type × Storage day	ns	ns	**	ns	**	**	*	ns	ns
Product variety × Storage day	**	**	**	**	**	**	**	**	*
Product variety × Culture type × Storage day	**	**	**	**	**	**	ns	ns	ns

*Note:* Lowercase letters indicate significant differences between groups.

Abbreviations: AMK—100% reconstituted milk, 1:9 K–90% reconstituted milk plus 10% chestnut milk; 4:1 K–80% reconstituted milk plus 20% chestnut milk; 7:3 K—70% reconstituted milk plus 30% chestnut milk; 3:2 K—60% reconstituted milk plus 40% chestnut milk; 1:1 K—50% reconstituted milk plus 50% chestnut milk; CMK—100% chestnut milk; ns, non‐significant *p* > 0.05.

** *p* ≤ 0.01.

* *p* ≤ 0.05.

### 3.4 Lipid Quality Indices of Samples

The lipid quality indices, based on the fatty acid profiles of the samples, are shown in Table 7. Statistical analysis revealed that chestnut milk had a significant effect (*p* ≤ 0.01) on the lipid quality indices. The sample (CMK) with 100% chestnut milk had the highest values for the MUFA/SFA, PUFA/SFA, and DFA indices, as well as the highest h/H and HPI values. Conversely, it had the lowest AI and TI values. The sample with 20% chestnut milk (4:1K) had significantly lower (*p* ≤ 0.01) mean values for MUFA/SFA, PUFA/SFA, DFA, h/H, and HPI ratios, and higher AI and TI. Lower AI and TI indices are closely associated with lower levels of SFAs and higher levels of PUFA, suggesting higher nutritional quality and a lower risk of cardiovascular disease. The PUFA/SFA ratio is above 0.45, which reflects a positive health effect (Guo et al. 2025). This study detected the PUFA/SFA values above 1, except for the 20% chestnut milk sample. Culture type significantly influenced lipid quality indices (*p* ≤ 0.01). The samples fermented with a commercial starter culture showed higher MUFA/SFA, PUFA/SFA, DFA, h/H, and HPI values, whereas those fermented with kefir grain showed slightly higher TI values. Significant differences (*p* ≤ 0.01) in lipid quality indices, except MUFA/SFA and HPI values, were observed across storage days. Greater PUFA/SFA, DFA, and h/H values were calculated at the beginning of storage, whereas AI and TI values were slightly higher on the final day of storage. The interactions of ‘product variety × culture type’, ‘product variety × storage day’, and ‘product variety × culture type × storage day’ were determined statistically significant (*p* ≤ 0.01) for all values. The interactions of ‘culture type × storage day’ were determined to be statistically significant for PUFA/SFA, h/H, HPI, and AI values (*p* ≤ 0.01). The results of the present study align with those of Abbas et al. (2021), who investigated the impact of walnut addition to processed cheese on its lipid nutritional value. They indicated that cheese with walnuts had higher PUFA/SFA, PUFA/MUFA, and DFA and lower w6/w3 values than cheese without walnuts. The AI and TI of walnut‐processed cheeses ranged from 0.80 to 1.21 and 0.32 to 0.50, respectively. The walnut addition decreased the AI and TI values of the samples. Kasapidou et al. (2023) reported that the AI, TI, and h/H ratios of almond‐based milk ranged from 0.15 to 0.19, 0.23 to 0.31, and 9.43 to 11.47, respectively. In comparison, coconut‐based milk exhibited AI, TI, and h/H values of 5.55, 2.85, and 0.53, respectively, whereas peanut‐based milk showed values of 0.19, 0.32, and 8.11, respectively. The AI, TI, and h/H indices reported in the study for almond‐, oat‐, and peanut‐based milk were found to agree with the values obtained in the present study. Laučienė et al. (2019) investigated lipid quality indices of fresh cheese manufactured with mesophilic lactic acid bacteria (LAB, including *Lactobacillus lactis* subsp. *cremoris*, subsp. *lactis*, and subsp. *lactis* biovar *diacetylactis*, as well as *Leuconostoc* subsp.) in both summer and winter. They determined that the indices of cheese samples in summer and winter were 0.49 and 0.35 for h/H, 38.26 and 31.91 for DFA, 3.46 and 4.23 for AI, and 3.11 and 3.93 for TI, respectively. It was reported that the AI and TI indices of probiotic kefirs produced with *L. acidophilus* La‐5 and *Bifidobacterium animalis* subsp. *lactis* BB‐12 and enriched with walnut oil (0.03%; w/w) ranged between 2.7–3.2 and 3.2–3.9, respectively, throughout 14 days of storage, indicating that the formulation and storage duration had no significant interactive effect on these indices (Turek and Wszołek 2022). Paszczyk and Tońska (2025) found that yogurts enriched with hemp seed had the highest proportion of DFA (53.86%), and that the AI and TI indices were markedly reduced in yogurts enriched with chia, hemp, quinoa, and oat bran, which had lower values than the control. PUFA/SFA, AI, and TI values of milk fermented with different kefir grains were reported by Vieira et al. (2015) to range from 0.02 to 0.04, 3.25 to 6.53, and 4.57 to 9.36, respectively. In addition, researchers highlighted that AI and TI values decreased, whereas PUFA/SFA ratio and HH index increased during 14‐d storage. Antunes et al. (2024) also compared lipid quality indices among plant‐based milk alternatives (soya, oat, rice, almond, coconut, and hazelnut) and dairy milk. Hazelnut milk exhibited the highest h/H ratio (15.2), while coconut milk and semi‐skimmed milk showed the lowest values (~0.4). Coconut milk also had the highest AI (11.4) and TI (7.8) indices, exceeding those of dairy milks. They highlighted substantial differences in lipid quality among soya, oat, rice, almond, coconut, and hazelnut‐based milks, largely influenced by their botanical origin. Dimitrova et al. (2022) reported that, for kefir produced from goat's milk of the ‘Bulgarian White Dairy’ breed and its crosses with ‘Toggenburg’ and Anglo‐Nubian, the lipid quality indices on the 14th day of storage varied as follows: AI ranged from 3.11 to 4.52, TI from 2.14 to 3.27, and h/H ratio from 0.42 to 0.56. In a similar study, Vukić et al. (2019) reported that the AI and TI indices of the fermented milk samples ranged from 2.14 to 2.39 and from 2.91 to 3.18, respectively, in relation to the effect of non‐conventional starter culture on the lipid nutritional quality of fermented dairy products. These changes in lipid quality indices (AI, TI, h/H, and HPI) clearly demonstrate that the addition of chestnut milk structurally transforms the atherogenic and thrombogenic potential of the fermented matrix. The low AI and TI values, along with the high h/H ratios observed in CMK samples, can be explained not only by the reduction in SFAs but also by the protective effect of chestnut PUFA within the matrix. From a biochemical perspective, the decrease in AI value indicates that the pro‐atherogenic effects of lauric (C12:0), myristic (C14:0), and palmitic (C16:0) acids are suppressed by high levels of oleic and linoleic acids derived from chestnuts. This theoretically means a reduction in the risk of lipid peroxidation on the cardiovascular system. The higher h/H and HPI values provided by commercial starter cultures suggest that these strains exhibit stronger antioxidant capacity against lipid oxidation or that their lipolytic activities selectively stabilize hypocholesterolemic fatty acids. The increase in AI and TI indices during storage indicates that the lipid profile shows a limited shift toward SFAs, due to the oxidative sensitivity of PUFA and changes in microbial metabolism. Nevertheless, the PUFA/SFA ratio remains above the critical threshold of 0.45 (Guo et al. 2025) across all formulations, demonstrating that the product maintains its lipid quality throughout its shelf life. The significant interaction between ‘product variety × culture type × storage day’ reveals that lipid quality indices are not static indicators; rather, they are dynamically shaped by strain‐specific enzymatic activities of the kefir microbiota during storage. In this context, time‐dependent changes observed in the indices are attributed to matrix reorganization driven by pH‐dependent structural transformations and protein‐lipid‐polysaccharide interactions, rather than to uncontrolled lipid degradation. In conclusion, the findings reveal that the addition of chestnut milk dynamically modulates the lipid profile and quality indices in mixed fermented systems; this interaction functions as a fundamental mechanism that enhances the nutritional functionality and technological stability of the product by integrating with culture‐specific metabolic pathways and matrix reorganization.

**TABLE 7:** Lipid quality indices of the samples.

Product varieties	MUFA/SFA	PUFA/SFA	DFA	h/H	HPI	AI	TI
AMK	0.87^c^	1.08^d^	65.27^e^	2.30^d^	1.43^e^	0.77^b^	0.91^b^
1:9K	0.93^b^	1.10^cd^	65.44^de^	2.37^c^	1.65^c^	0.62^d^	0.86^c^
4:1K	0.70^e^	0.65^e^	57.68^f^	1.55^f^	1.01^f^	1.00^a^	1.24^a^
7:3K	0.86^c^	1.21^b^	66.88^c^	2.46^b^	1.64^c^	0.64^c^	0.82^d^
3:2K	0.83^d^	1.13^c^	66.26^cd^	2.22^e^	1.57^d^	0.63^cd^	0.88^c^
1:1K	0.91^b^	1.25^b^	68.57^b^	2.46^b^	1.77^b^	0.57^e^	0.81^d^
CMK	0.97^a^	1.48^a^	71.34^a^	2.85^a^	1.91^a^	0.53^f^	0.70^e^
*Culture types*							
Grain	0.84^b^	1.10^b^	65.66^b^	2.27^b^	1.55^b^	0.69^a^	0.90^a^
Commercial	0.89^a^	1.15^a^	66.17^a^	2.36^a^	1.60^a^	0.68^b^	0.87^b^
*Storage days*							
1st day	0.87^a^	1.16^a^	66.49^a^	2.41^a^	1.56^a^	0.67^b^	0.84^b^
21st day	0.87^a^	1.10^b^	65.34^b^	2.23^b^	1.58^a^	0.69^a^	0.92^a^
*ANOVA*							
Product variety	**	**	**	**	**	**	**
Culture type	**	**	*	**	**	*	**
Storage day	ns	**	**	**	ns	**	**
Product variety × Culture type	**	**	**	**	**	**	**
Culture type × Storage day	ns	**	ns	**	**	*	ns
Product variety × Storage day	**	**	**	**	**	**	**
Product variety × Culture type × Storage day	**	**	**	**	**	**	**

*Note:* Lowercase letters indicate significant differences between groups.

Abbreviations: AMK—100% reconstituted milk, 1:9 K–90% reconstituted milk plus 10% chestnut milk; 4:1 K–80% reconstituted milk plus 20% chestnut milk; 7:3 K—70% reconstituted milk plus 30% chestnut milk; 3:2 K—60% reconstituted milk plus 40% chestnut milk; 1:1 K—50% reconstituted milk plus 50% chestnut milk; CMK—100% chestnut milk; ns, non‐significant *p* > 0.05.

** *p* ≤ 0.01.

* *p* ≤ 0.05.

### 3.5 Principal Component Analysis

**FIGURE 3:**
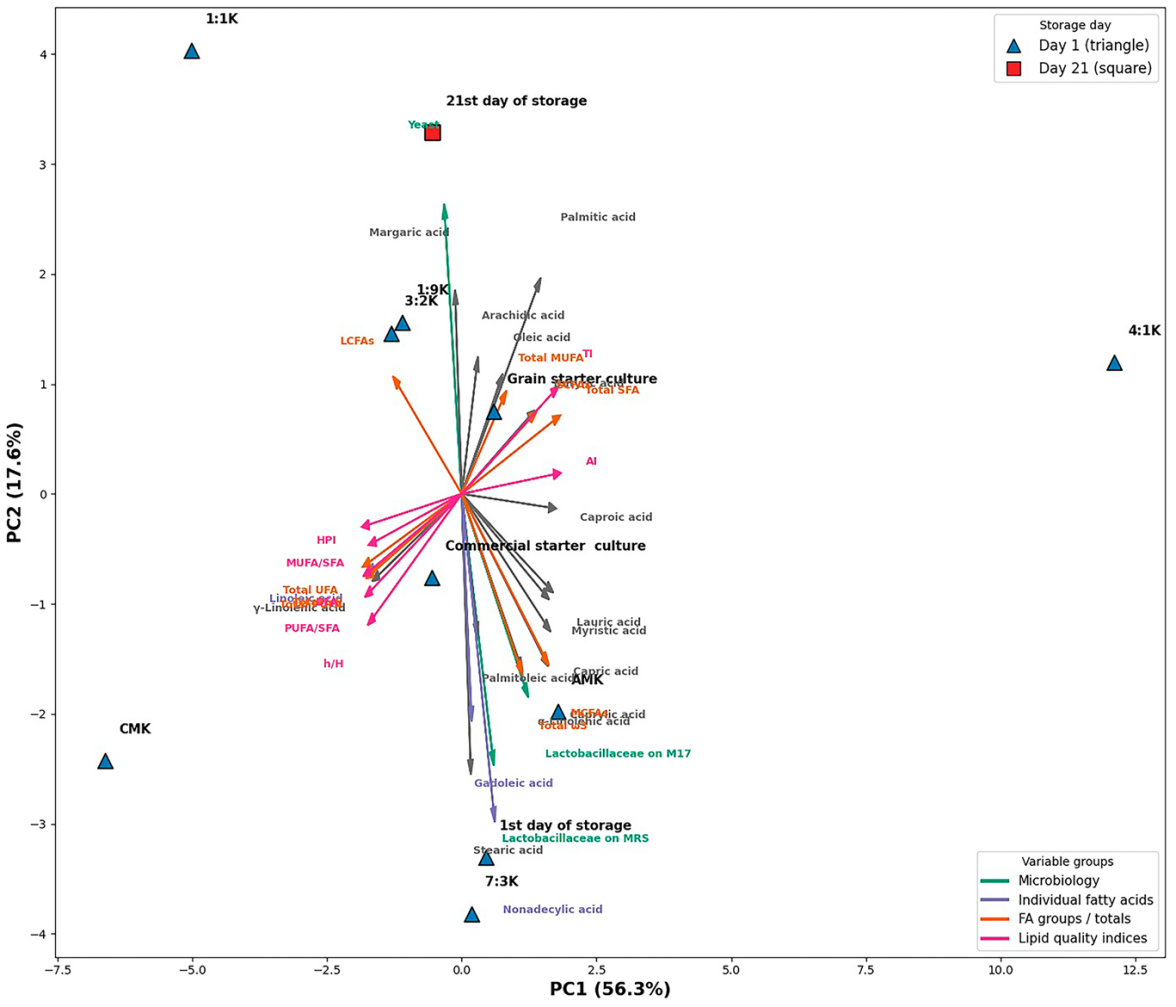
PCA biplot based on the parameters analyzed of the samples.

The principal component analysis (PCA) biplot in Figure 3 shows that variation among samples is largely explained by PC1 (56.3%) and PC2 (17.6%), which together account for 73.9% of the total variance. The separation observed along PC1 is primarily driven by formulation‐dependent lipid composition and lipid quality indices, with samples aligning with nutritionally favorable indices such as PUFA/SFA, MUFA/SFA, h/H, and HPI as the proportion of chestnut milk increases. This situation reveals that, particularly, the CMK (100% chestnut milk) sample is positioned in the negative direction of PC1, exhibiting a strong relationship with UFA fractions and cardioprotective lipid quality indicators. In contrast, the positioning of 4:1K (20% chestnut milk) and AMK (100% reconstituted milk) samples in the positive direction of PC1 indicates that these samples cluster with lipid parameters associated with higher atherogenic and thrombogenic potential, such as total SFA, AI, and TI. This separation clearly supports that chestnut milk acts not just as a volume substitute in the fermented matrix but as a functional component that transforms the lipid profile and quality indices. The PC2 axis mainly reflects the effects of storage duration and microbial composition. Specifically, day 1 samples are positioned in the negative direction of PC2 in relation to Lactobacillaceae (MRS and M17) counts and certain long‐chain fatty acids, while day 21 samples are positioned in the positive direction in association with yeast counts and short‐medium chain fatty acids. This indicates that changes in the lipid profile during storage reflect a dynamic process driven by microbial metabolic activity and matrix reorganization rather than static oxidative degradation. When examining the orientations of fatty acid vectors, the concentration of palmitic, oleic, and arachidic acids in the positive direction of PC1 indicates that these acids are more associated with animal milk‐dominant formulations; whereas linoleic, α‐linolenic, and total PUFA fractions align with samples high in chestnut milk content, revealing the determinative effect of plant lipids in the fermented matrix. The clustering of arrows representing lipid quality indices (PUFA/SFA, h/H, HPI) in the same region confirms that these indices are strongly positively correlated with each other and are consistently separated by PCA. Overall, the PCA biplot analysis reveals that lipid quality results in this study are primarily determined by formulation (chestnut milk ratio) and secondarily by microbial activity and storage duration. These findings strongly support the view that lipid quality in mixed fermented systems cannot be reduced to fatty acid composition alone; rather, the microbial ecosystem, protein‐lipid‐polysaccharide interactions, and matrix dynamics that develop during storage should be evaluated together. In a relevant study, Antunes et al. (2024) conducted a PCA to assess differences in lipid profiles among milk alternatives made from milk, soya, oat, rice, almond, coconut, and hazelnut.

## 4 Conclusion

This study demonstrated that incorporating chestnut milk into kefir formulations significantly affects the fatty acid composition and improves the lipid quality profile, depending on the ratio, culture type, and storage duration. In particular, kefir made with 100% chestnut milk exhibited the highest PUFA/SFA, DFA, h/H, and HPI values, highlighting its potential as a functional, low‐fat fermented product. Commercial starter culture further enhanced the nutritional quality of lipid fractions by preserving bioactive fatty acids, such as omega‐3 and conjugated linolenic acids, during storage. From an industrial perspective, developing plant‐based dairy alternatives with improved health‐promoting lipid indices aligns with the growing consumer demand for functional and sustainable foods. Chestnut milk, naturally low in fat but rich in essential fatty acids and bioactives, offers a promising substrate for novel kefir production with enhanced nutritional functionality. Future studies should focus on optimizing fermentation parameters and scaling up production processes to enhance efficiency and productivity. Moreover, in vivo investigations are necessary to validate the health benefits of chestnut milk‐based kefir, particularly in relation to lipid metabolism, cardiovascular protection, and gut health. Integrating metabolomic, sensory, and clinical perspectives will further enhance the applicability of this product in the functional dairy sector.

## Author Contributions

**Buse Usta‐Gorgun:** writing – investigation, methodology, formal analysis, data curation, original draft, visualization, validation. **Lutfiye Yilmaz‐Ersan:** supervision, project administration, investigation, conceptualization, resources, writing – original draft, writing – review and editing.

## Funding

This work was supported by the Scientific and Technological Research Council of Turkey (TUBITAK 118O428) and Bursa Uludag University Scientific Research Projects Unit (BAP, DDP (Z)‐2019/8).

## Conflicts of Interest

The authors declare no conflicts of interest.

## Data Availability

The data that support the findings of this study are available from the corresponding author upon reasonable request.
